# The linear association between high-density lipoprotein cholesterol and diabetic retinopathy : a cross-sectional study in diabetic patients

**DOI:** 10.1007/s40200-025-01728-z

**Published:** 2025-10-18

**Authors:** Zhuolin Xie, Xiangxia Luo, Di Ling, Dongpeng Zhang, Xinglin Chen, Dinghua Zhang, Juan Ling

**Affiliations:** 1 Gansu Provincial Hospital of TCM, Lanzhou, Gansu Province 730050 China; 2 The Third People’s Hospital of Gansu Province, Lanzhou, Gansu Province 730050 China; 3 Department of Geriatrics, Union Hospital, Tongji Medical College, Huazhong University of Science and Technology, Wuhan, China; 4 Department of Epidemiology and Biostatistics, Empower U, X&Y Solutions Inc., Boston, USA; 5 Clinical College of Chinese Medicine, Gansu University of Chinese Medicine , Lanzhou, Gansu Province 730030 China; 6 Gansu Provincial Hospital, Lanzhou, 730030 China

**Keywords:** High-density lipoprotein, Cholesterol, Diabetic retinopathy

## Abstract

**Background:**

Diabetic retinopathy (DR) is a major microvascular complication of diabetes mellitus and a leading cause of visual impairment. While dyslipidemia has been implicated in DR pathogenesis, the relationship between high-density lipoprotein cholesterol (HDL-C) and DR remains controversial. This study aimed to investigate the association between HDL-C levels and DR prevalence in diabetic patients type 2 diabetes mellitus (T2DM).

**Methods:**

This study is the second analysis based on a cross-sectional studv. A total of 2001 (858 men and 1143 women) diabetic patients who visited the diabetic clinic in the Internal Medicine out-patient departments of two hospitals in southern Taiwan between April 2002 and November 2004. Demographic and clinical data were collected, and serum HDL-C levels were measured. The association between HDL-C and DR was analyzed using multivariate logistic regression, accounting for potential confounders. Additionally, we explored the potential correlation between HDL-C and DR through a smooth curve fitting approach, utilizing a generalized additive model (GAM). and a generalized additive model (GAM).

**Results:**

Among the 2001 participants, 701 (35.0%) were diagnosed with DR. Our findings demonstrated a significant inverse linear relationship between HDL-C levels and the presence of DR. Higher HDL-C was inversely associated with diabetic retinopathy (DR). In continuous analyses, each 10 mg/dL increase in HDL-C corresponded to lower odds of DR across all models, including Model 3 (OR 0.92; 95% CI 0.84–0.99; *P* = 0.027).Additionally, analysis of HDL-C levels by tertiles revealed that participants in the highest tertile (53.0–99.0 mg/dL) had a lower prevalence of DR, with an OR of 0.78 (95% CI: 0.62–0.97; *P* = 0.029) in Model 1; this association was borderline in Model 2 (OR: 0.80; 95% CI: 0.63–1.01; *P* = 0.055) and non-significant in Model 3 (OR: 0.86; 95% CI: 0.66–1.09; *P* = 0.209). In categorical analyses (reference: ≤40 mg/dL), participants with HDL-C ≥ 60 mg/dL exhibited a significantly lower prevalence of DR in Model 1 (OR: 0.73; 95% CI: 0.56–0.97; *P* = 0.028), a borderline association in Model 2 (OR: 0.76; 95% CI: 0.57–1.01; *P* = 0.059), and a non-significant association in Model 3 (OR: 0.83; 95% CI: 0.61–1.14; *P* = 0.259).

**Conclusions:**

This study provides evidence of a linear association that elevated HDL-C levels are associated with decreased odds of DR in diabetic patients. Future research should further focus on elucidating the mechanisms underlying this association and its implications for therapeutic strategies.

**Supplementary Information:**

The online version contains supplementary material available at 10.1007/s40200-025-01728-z.

## Introduction

Diabetic retinopathy (DR), a microvascular complication of diabetes mellitus, is the primary cause of vision loss in the working-age population worldwide [1]. With the global prevalence of diabetes escalating, the incidence of DR is anticipated to increase concomitantly [2]. DR is characterized by retinal vascular damage, which can culminate in severe visual impairment if unaddressed. According to the latest epidemiological data, approximately 420 million people worldwide have diabetes, with about 30% developing DR, a figure that is even higher among those with a longer duration of diabetes [3]. DR significantly impacts patients’ quality of life and imposes a substantial economic burden on society. Its etiology involves multiple factors, including hyperglycemia, hypertension, and hyperlipidemia, while the pathogenesis is closely related to inflammation, oxidative stress, and endothelial dysfunction in the retinal microvasculature [4]. As diabetes continues to rise globally, understanding the underlying mechanisms and risk factors that contribute to the development and progression of DR is essential. Among these factors, dyslipidemia, particularly alterations in high-density lipoprotein cholesterol (HDL-C) levels, has garnered attention for its potential role in the pathophysiology of DR. Research suggests that HDL-C not only plays a crucial role in lipid metabolism but may also exert protective effects through anti-inflammatory and antioxidant mechanisms that are vital for retinal health. Thus, investigating the relationship between HDL-C levels and DR could provide insights into the disease’s mechanisms and open avenues for novel therapeutic strategies.

HDL-C has traditionally been recognized for its protective role in cardiovascular health; however, its association with diabetic retinopathy remains complex and controversial.Recent studies have shown that dyslipidemia, particularly low levels of HDL-C, is prevalent among individuals with diabetic patients and is associated with the progression of diabetic retinopathy. For instance, one study demonstrated that lower HDL-C levels correlated with the severity of DR among T2DM patients, suggesting that HDL-C may play a critical role in the pathophysiology of DR [5]. Conversely, other research indicates that elevated levels of HDL-C may not confer the expected protective benefits against DR, highlighting the need for further investigation into the functional properties of HDL-C, rather than just its concentration in serum [6]. Observational studies have consistently highlighted the relationship between HDL-Clevels and DR. For instance, a study involving 1,450 patients with type 2 diabetes found that those diagnosed with DR exhibited a significantly elevated uric acid to HDL-C ratio compared to those without DR, suggesting that HDL-C may play a protective role against the development of DR [7]. Another investigation indicated that lower HDL levels were associated with increased severity of DR, emphasizing the potential role of dyslipidemia in the progression of diabetic complications [5]. Furthermore, a study conducted in Saudi Arabia revealed that while triglyceride and low-density lipoprotein cholesterol levels were significantly associated with DR progression, HDL-C did not show a significant correlation, suggesting that low HDL-C may not be a direct risk factor but could be indicative of overall dyslipidemia [8]. These findings collectively underscore the need for further research to clarify the complex association between HDL-C levels and the DR, as well as the potential for HDL-C to serve as a biomarker for early detection and intervention strategies.

In summary, the relationship between HDL-C and DR is multifaceted, involving metabolic, inflammatory, and oxidative stress pathways. Understanding this relationship not only enhances our comprehension of DR’s pathophysiology but also paves the way for innovative therapeutic approaches that target lipid metabolism as a means to combat this debilitating complication of diabetes. As research continues to evolve, it is crucial to establish clear clinical guidelines that incorporate lipid management into the standard care protocols for diabetic patients. Moreover, emerging evidence suggests that HDL-C may have direct implications for retinal health. In this cross-sectional study, we aim to investigate the association between HDL-C and DR in diabetic patients. We will explore the potential influence of HDL-C on the development and progression of DR, taking into account the complexity of their relationship and the possible interplay with demographic, metabolic, and genetic factors. Our findings aspire to contribute to the current scientific understanding of HDL-C’s role in DR and offer insights that may guide clinical practice and future therapeutic strategies.

## Methods

### Study population

In this secondary analysis, we utilized data derived from the study conducted by Chen SC et al. [9], published in the esteemed journal PloS One (10.1371/journal.pone.0134718). This dataset was made freely available for download, adhering to principles of open-access research. The investigation carried out by Chen SC et al. represented a comprehensive survey conducted across diabetes clinics within the Internal Medicine outpatient departments of two hospitals located in southern Taiwan, covering the time frame from April 2002 to November 2004. The diagnostic criteria for diabetes include: random plasma glucose ≥ 11.1 mmol/L (200 mg/dL) with symptoms, fasting plasma glucose ≥ 7.0 mmol/L (126 mg/dL), 2-h plasma glucose ≥ 11.1 mmol/L (200 mg/dL) during an oral glucose tolerance test, or HbA1c ≥ 6.5% (48 mmol/mol). A second test on a different day is required for confirmation if asymptomatic [10]. The study excluded patients with type 1 diabetes mellitus (defined as those presenting with diabetic ketoacidosis, acute hyperglycemia symptoms accompanied by heavy ketonuria [≧ 3], or those who required continuous insulin therapy in the year following diagnosis), as well as patients undergoing dialysis or with an estimated glomerular filtration rate (eGFR) of less than 15 ml/min/1.73 m², and patients who had received a renal transplant. After careful selection, the final study sample included 2001 individuals with a mean age of 64.1 ± 11.3 years, consisting of 858 males and 1143 females.

### Ethics statement

The original study has already obtained the necessary Ethics Statement and the study was conducted in accordance with the Declaration of Helsinki, adhering to both international ethical standards and local regulations of previously study [9]. This study was conducted in accordance with the Declaration of Helsinki and adhered to international ethical guidelines and local regulatory requirements. The study protocol was approved by the Institutional Review Board of Kaohsiung Medical University Hospital (approval number: KMUHIRB-E-20150029). Written informed consent was obtained from all participants prior to their inclusion, including consent for the publication of their anonymized clinical data.

### Variables

Demographic and clinical characteristics were obtained from medical records and patient interviews, including age, gender, and comorbid conditions. Height and weight were measured in all participants using a calibrated digital scale and a stadiometer. Participants were instructed to stand straight with their feet together and their backs against the stadiometer. Weight was recorded to the nearest 0.1 kg, and height was measured to the nearest 0.1 cm. Body mass index (BMI) was calculated as weight (kg) divided by height squared (m²). Blood pressure was measured using an automatic oscillometric device (e.g., VP1000; Colin Co. Ltd., Komaki, Japan) after participants had rested for at least five minutes in a seated position. Measurements were taken in both arms; the average of two readings from the arm with the higher pressure was used for analysis. Abdominal circumference was measured at the level of the iliac crest using a flexible measuring tape. Participants were instructed to relax and exhale normally during the measurement. The measurement was taken at the end of a normal expiration and recorded to the nearest 0.1 cm. The ankle-brachial index (ABI) values were measured using an ABI-form device (VP1000; Colin Co. Ltd., Komaki, Japan), which automatically and simultaneously measures blood pressure in both arms and ankles utilizing an oscillometric method [11, 12]. The ABI was calculated as the ratio of the ankle systolic blood pressure to the arm systolic blood pressure. Each patient underwent a single ABI measurement. Peripheral artery disease (PAD) was defined as an ABI < 0.9 or ≥ 1.3 in either leg.

Serum creatinine was measured using the compensated Jaffé (kinetic alkaline picrate) method on a Roche/Integra 400 Analyzer, with calibration traceable to isotope-dilution mass spectrometry [13]. Estimated glomerular filtration rate (eGFR) was calculated using the 4-variable Modification of Diet in Renal Disease (MDRD) equation [14]. rine albumin and creatinine were analyzed from spot urine samples using an autoanalyzer (COBAS Integra 400 Plus; Roche Diagnostics, North America). DR was classified as as no retinopathy and retinopathy.

Ischemic heart disease (IHD), also known as coronary artery disease (CAD), is primarily characterized by a reduced blood supply to the heart muscle due to coronary artery obstruction. The clinical manifestations of ischemic heart disease are diverse, with the most common symptom being chest pain or discomfort, often described as a feeling of pressure or squeezing [15, 16]. The World Health Organization (WHO) defines stroke as a clinical syndrome characterized by the rapid onset of focal neurological deficits that last for more than 24 h or lead to death, with no apparent cause other than vascular origin [17].

### Statistical analysis

The clinical characteristics of the participants were categorized into three groups: no diabetic retinopathy (NDR), non-proliferative diabetic retinopathy (NPDR), and proliferative diabetic retinopathy (PDR) according to the presence or absence of DR. NCEP-ATP III criteria of HDL-C levels as follows: (1) Low HDL-C: HDL-C < 40 mg/dL (< 1.03 mmol/L); (2) Normal/Intermediate HDL-C: HDL-C between 40 and 59 mg/dL (1.03–1.55 mmol/L); (3) High HDL-C: HDL-C ≥ 60 mg/dL (−1.55 mmol/L). Continuous variables underwent normality tests (such as the Shapiro-Wilk test and the Kolmogorov-Smirnov test) and assessments of homogeneity of variance. For variables that met these assumptions, intergroup comparisons were conducted using one-way analysis of variance (ANOVA). If these assumptions were not satisfied, non-parametric tests, such as the Kruskal-Wallis test, were employed [18, 19].

Categorical variables were expressed as counts and percentages, whereas continuous variables were reported as either means with standard deviations (SD) or medians with interquartile ranges (25 th to 75 th percentiles), based on the data distribution. For continuous variables with abnormal distribution, data are presented in the form of “Median (Q1-Q3)” with p-values obtained by Mann-Whitney U test. For categorical variables, data are presented as in the form of “sample size (%)” with p-value obtained by Chi-square test.The relationship between diabetic retinopathy (DR) and high-density lipoprotein cholesterol (HDL-C) levels was investigated using multivariate logistic regression and smooth curve fitting, while adjusting for relevant clinical covariates. To further elucidate this relationship, we employed generalized additive models (GAMs) along with smooth curve fitting techniques. This analytical approach is well-suited for exploring potential associations between various factors and disease outcomes. Besides, subgroup analyses and interaction tests were performed to further examine the association between the HDL-C and DR.

In this study, we evaluated multicollinearity among the independent variables to ensure the stability and reliability of the model results. We employed the Variance Inflation Factor (VIF) as the detection tool. Each independent variable’s VIF value was compared against a threshold (VIF ≥ 5) to identify and remove highly collinear variables [20, 21]. Through this assessment process, we retained important variables relevant to the research outcomes, thereby enhancing the model’s effectiveness and interpretability. The sofware for statistical analysis of the data was EmpowerStats version 4.1 (www.empowerstats.net, X&Y solutions, Inc. Boston, Massachusetts) and the R language package version 4.2.0 (The R Foundation; http://www.r-project.org; version 4.2.0).

### DR confirmation

Confirmation of DR was conducted by certified ophthalmologists through comprehensive eye examinations. Patients underwent fundus photography and optical coherence tomography (OCT) to identify characteristic retinal lesions, including microaneurysms, hemorrhages, and exudates. The diagnosis was established based on the International Clinical Diabetic Retinopathy Disease Severity Scale, which confirmed the presence or absence of DR [22].

## Result

A total of 2001 diabetic patients were included in this study and the comparison of baseline characteristics between NDR, non-proliferative diabetic retinopathy (NPDR) and proliferative diabetic retinopathy (PDR) were shown in Table 1.

**Table 1:** Baseline characteristics of the study participants (*n* = 2001)

Characteristics	NDR (*n* = 1300)	NPDR (*n* = 340)	PDR (*n* = 361)	*P*-value
Age(years)	63.3 ± 11.8	66.0 ± 10.5	64.8 ± 9.9	< 0.001
BMI(kg/m²)	25.8 ± 3.6	25.7 ± 3.5	26.1 ± 3.3	0.394
DBP(mmHg)	77.8 ± 11.1	77.5 ± 11.6	77.9 ± 11.8	0.869
HbA1c(%)	7.6 ± 1.7	7.6 ± 1.6	7.9 ± 1.5	0.01
AC(cm)	148.0 ± 51.6	147.0 ± 46.9	151.9 ± 55.0	0.374
Creatine(mg/dL)	186.5 ± 38.3	183.8 ± 40.0	184.5 ± 38.6	0.402
Triglycerides(mg/dL)	154.9 ± 136.2	158.7 ± 146.6	151.4 ± 121.2	0.774
LDL(mg/dL)	104.8 ± 28.5	102.7 ± 29.3	104.0 ± 27.6	0.466
HDL(mg/dL)	50.1 ± 13.2	48.6 ± 13.4	48.4 ± 11.9	0.025
Creatine(mg/dL)	1.0 ± 0.4	1.1 ± 0.4	1.2 ± 0.5	< 0.001
eGFR(mL/min/1.73 m²)	70.4 ± 19.4	65.7 ± 19.6	65.1 ± 20.1	< 0.001
ABI	1.1 ± 0.1	1.1 ± 0.1	1.1 ± 0.1	0.599
Sex				0.409
Male	748 (57.5%)	199 (58.5%)	195 (54.0%)	
Female	552 (42.5%)	141 (41.5%)	166 (46.0%)	
HDL tertile				0.18
Low(15.0-42.0 mg/dL)	404 (31.2%)	127 (37.5%)	127 (35.4%)	
Middle(43.0-52.0 mg/dL)	421 (32.5%)	99 (29.2%)	115 (32.0%)	
High(53.0–99.mg/dL0)	469 (36.2%)	113 (33.3%)	117 (32.6%)	
HDL categorical				0.175
≦ 40 mg/dL	322 (24.9%)	99 (29.2%)	95 (26.5%)	
40-59 mg/dL	688 (53.2%)	179 (52.8%)	201 (56.0%)	
≧ 60 mg/dL	284 (21.9%)	61 (18.0%)	63 (17.5%)	
ACR > 30 mg/g				< 0.001
No	911 (70.1%)	207 (60.9%)	185 (51.2%)	
Yes	389 (29.9%)	133 (39.1%)	176 (48.8%)	
Ischemic Heart Disease				0.025
No	1102 (84.8%)	269 (79.1%)	293 (81.2%)	
Yes	198 (15.2%)	71 (20.9%)	68 (18.8%)	
Stroke				0.001
No	1249 (96.1%)	323 (95.0%)	330 (91.4%)	
Yes	51 (3.9%)	17 (5.0%)	31 (8.6%)	
β-blocker use (%)				0.173
No	1006 (77.7%)	259 (76.2%)	263 (73.1%)	
Yes	288 (22.3%)	81 (23.8%)	97 (26.9%)	
ACEI and/or ARB use (%)				< 0.001
No	381 (29.4%)	87 (25.6%)	59 (16.4%)	
Yes	913 (70.6%)	253 (74.4%)	301 (83.6%)	
Calcium channel blocker use (%)				< 0.001
No	848 (65.5%)	184 (54.1%)	181 (50.3%)	
Yes	446 (34.5%)	156 (45.9%)	179 (49.7%)	

Mean ± SD or Median (25th, 75th percentile) for continuous variables; P value was calculated by weighted chi-square test; *ABI *ankle-brachial index; *eGFR *estimated glomerular filtration rate; *ACR *albumin-to-creatinine ratio; *BMI *Body Mass Index; *DBP *Diastolic Blood Pressure

Table 2 presents the association between HDL-C and DR. In continuous analyses, we observed a significant inverse association across all models. Each 10 mg/dL increase in HDL-C was associated with lower odds of DR in Model 1 (OR: 0.91; 95% CI: 0.84–0.97; *P* = 0.007), Model 2 (OR: 0.91; 95% CI: 0.84–0.98; *P* = 0.014), and Model 3 (OR: 0.92; 95% CI: 0.84–0.99; *P* = 0.027). In tertile analyses (reference: low tertile, 15.0–42.0 mg/dL), the highest tertile (53.0–99.0 mg/dL) showed a significant inverse association with DR in Model 1 (OR: 0.78; 95% CI: 0.62–0.97; *P* = 0.029), which attenuated to borderline in Model 2 (OR: 0.80; 95% CI: 0.63–1.01; *P* = 0.055) and became non-significant in Model 3 (OR: 0.86; 95% CI: 0.66–1.09; *P* = 0.209). The middle tertile (43.0–52.0 mg/dL) did not differ significantly from the reference across models (Model 1: OR: 0.81; 95% CI: 0.64–1.01; *P* = 0.067; Model 2: OR: 0.82; 95% CI: 0.65–1.04; *P* = 0.101; Model 3: OR: 0.84; 95% CI: 0.65–1.06; *P* = 0.129).

**Table 2:** The association between HDL-C and DR

HDL-C (per 10 mg/dl)	Model 1 [OR (95% CI), *P*]	Model 2 [OR (95% CI), *P*]	Model 3 [OR (95% CI), *P*]
Overall	0.91 (0.84, 0.97), 0.007	0.91 (0.84, 0.98), 0.014	0.92 (0.84, 0.98), 0.027
HDL-C(Tertile)			
Low(15.0-42.0 mg/dL)	1.0	1.0	1.0
Middle(43.0-52.0 mg/dL)	0.81 (0.64, 1.01), 0.067	0.82 (0.65, 1.04), 0.101	0.84 (0.65, 1.06), 0.129
High(53.0–99.mg/dL)	0.78 (0.62, 0.97),0.029	0.80 (0.63, 1.01),0.055	0.86 (0.66, 1.09), 0.209
HDL-C categorical			
≦40 mg/dL	1.0	1.0	1.0
40-59 mg/dL	0.93 (0.74, 1.16),0.506	0.95 (0.76, 1.20), 0.688	1.01 (0.79, 1.30), 0.911
≧60 mg/dL	0.73 (0.56, 0.97),0.028	0.76 (0.57, 1.01), 0.059	0.83 (0.61, 1.14), 0.259

Model 1: no covariates were adjusted; Model 2: sex and Age were adjusted; Model 3: sex, age, BMI, LDL-C, triglycerides, DBP, Ischaemic Heart Disease, HbA1c(%), Creatine (mg/dL), ACEI and/or ARB use (%), β-blocker use (%), eGFR (mL/min/1.73m^2^), Calcium channel blocker use (%) and ABI

In categorical analyses (reference: ≤40 mg/dL), the 40–59 mg/dL group showed no significant association with DR (Model 1: OR: 0.93; 95% CI: 0.74–1.16; *P* = 0.506; Model 2: OR: 0.95; 95% CI: 0.76–1.20; *P* = 0.688; Model 3: OR: 1.01; 95% CI: 0.79–1.30; *P* = 0.911). Participants with HDL-C ≥ 60 mg/dL exhibited a significantly lower prevalence of DR in Model 1 (OR: 0.73; 95% CI: 0.56–0.97; *P* = 0.028), a borderline association in Model 2 (OR: 0.76; 95% CI: 0.57–1.01; *P* = 0.059), and a non-significant association in Model 3 (OR: 0.83; 95% CI: 0.61–1.14; *P* = 0.259).

Subgroup analyses and interaction tests were performed to further examine the association between the HDL-C and DR and evaluate potential factors that may influence this correlation (Table 3). Subgroups were categorized based on sex(Male or Female), age (19.8–59.5 years, 59.6–69.8 years and 69.9–96.4 years), BMI (15.4–24.1 kg/m², 24.2–27 kg/m² and 27.1–48.4 kg/m²), ACR > 30 mg/g (Yes or No), Stroke (Yes or No), chronic kidney disease (Yes or No), chronic obstructive pulmonary disease (Yes or No) and Ischaemic Heart Disease(Yes or No). No significant interactions were detected in these subgroups (*P* > 0.05).

**Table 3:** Subgroup analysis of the association between HDL-C and DR

Characteristic	No of participants	OR (95% CI)	*P* value	*P* for interaction
Stratified by Sex				0.76
male	1136	0.1 (0.0, 0.7)	0.016	
female	856	0.6 (0.1, 5.2)	0.648	
Stratified by Age				0.91
19.8–59.5 years	664	0.0 (0.0, 0.2)	0.002	
59.6–69.8 years	660	0.3 (0.0, 2.5)	0.235	
69.9–96.4 years	668	1.4 (0.1, 13.8)	0.799	
Stratified by BMI				0.72
15.4–24.1 kg/m²	663	0.1 (0.0, 0.7)	0.023	
24.2–27 kg/m²	666	0.1 (0.0, 1.5)	0.099	
27.1–48.4 kg/m²	663	1.2 (0.1, 14.0)	0.861	
Stratified by ACR > 30mg/g				0.64
Yes	1294	0.2 (0.0, 1.4)	0.106	
No	698	0.4 (0.0, 3.6)	0.406	
Stratified by Stroke				0.33
Yes	1894	0.2 (0.1, 0.9)	0.031	
No	98	1.8 (0.0, 465.3)	0.831	
Stratified by Ischaemic Heart Disease				0.45
Yes	1656	0.4 (0.1, 1.8)	0.23	
No	336	0.0 (0.0, 0.5)	0.02	

Above model adjusted for sex, age, BMI, LDL-C, triglycerides, DBP, Ischaemic Heart Disease, HbA1c(%), Creatine (mg/dL), ACEI and/or ARB use (%), β-blocker use (%), eGFR (mL/min/1.73m2), Calcium channel blocker use (%) and ABI

In each case, the model is not adjusted for the stratification variable

**Fig. 1:**
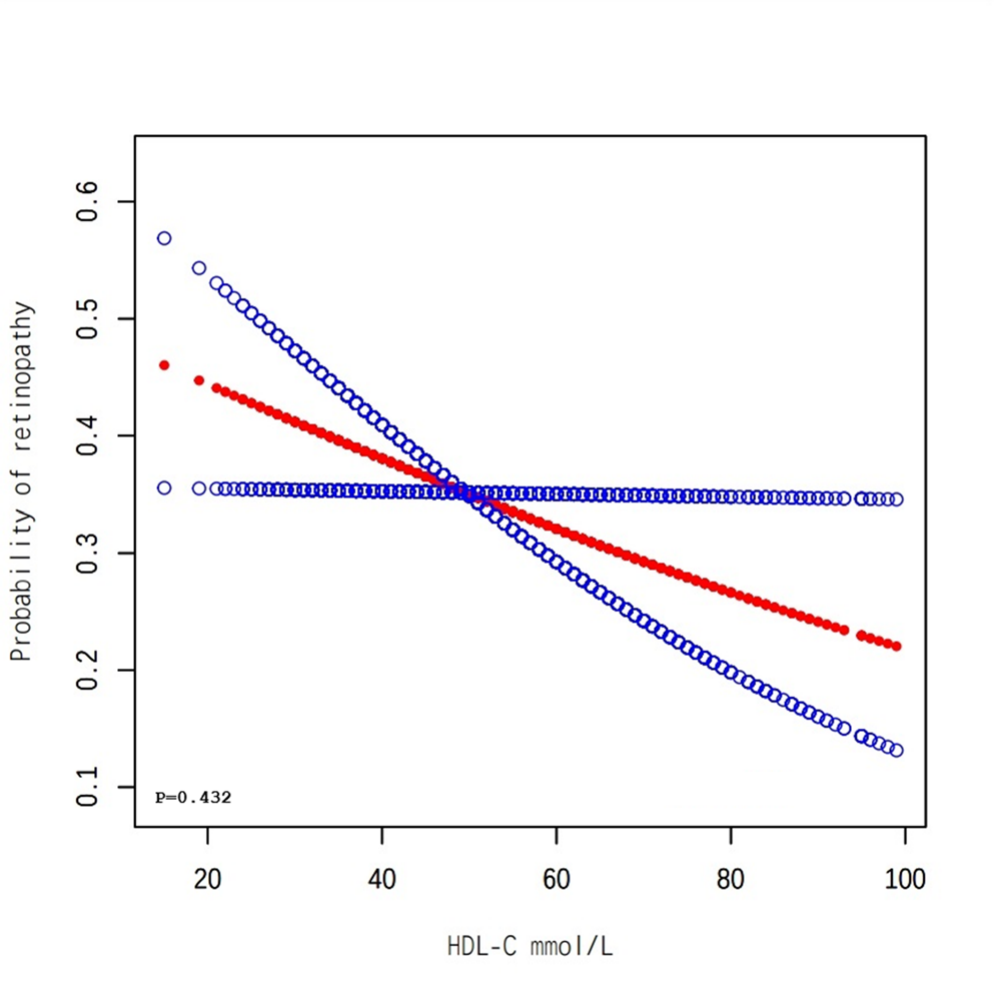
The association between HDL-C and DR. Red Line represents the smooth curve. Blue bands represent the 95% of confidence interval. Sex, age, BMI, LDL-C, triglycerides, DBP, Ischaemic Heart Disease, HbA1c(%), Creatine (mg/dL), ACEI and/or ARB use (%), β-blocker use (%), eGFR (mL/min/1.73m2), Calcium channel blocker use (%) and ABI were adjusted. Note: We employed generalized additive models (GAMs) to A generalized additive model (GAM) was used to test the non-linear relationship between DR and HDL-C and to create the smooth curve plots and the p-value for non-linearity in Figures 1, 2, 3, and 4 represents the value of the likelihood ratio test for threshold effects

**Fig. 2:**
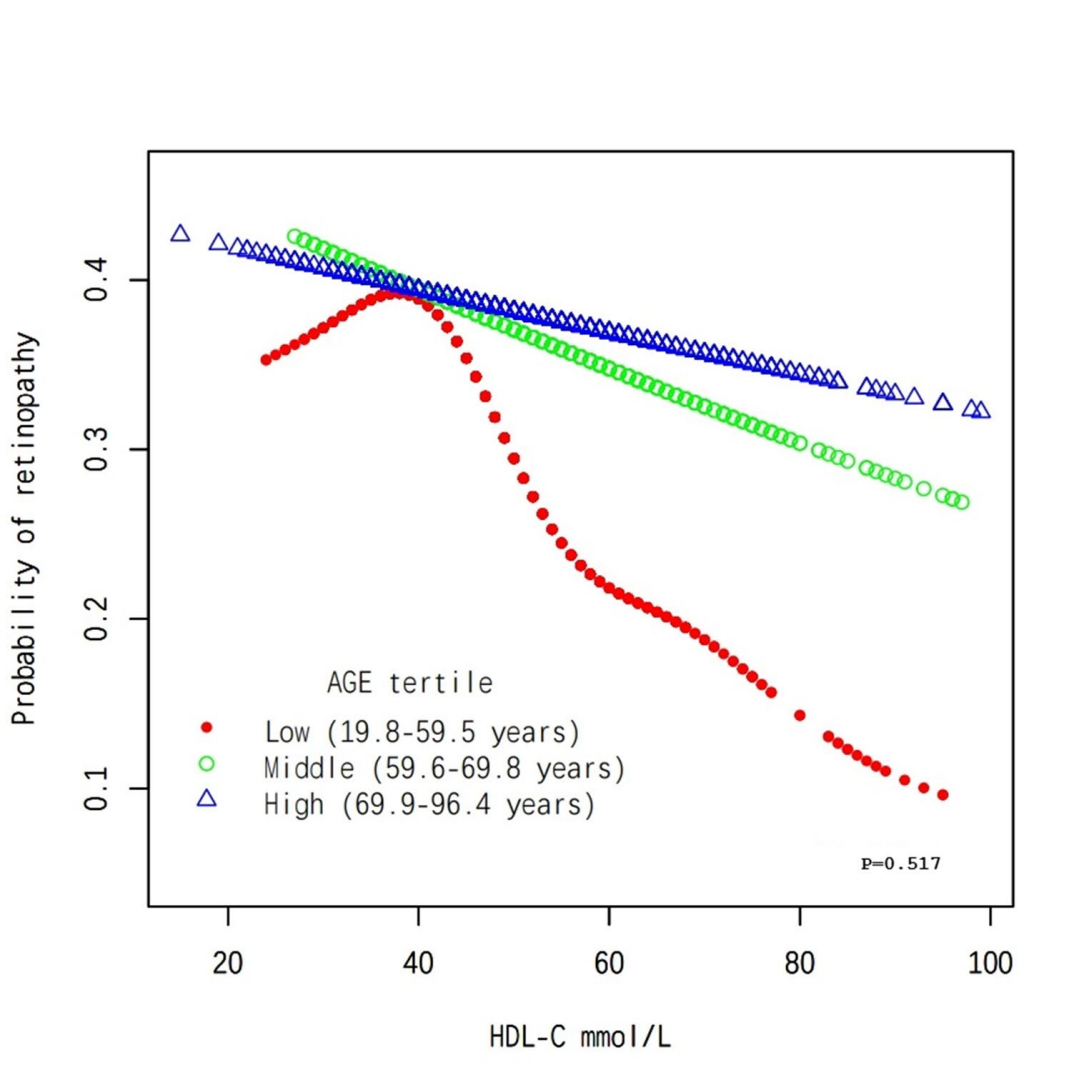
Subgroup analysis stratified by age. Sex, BMI, LDL-C, triglycerides, DBP, Ischaemic Heart Disease, HbA1c(%), Creatine (mg/dL), ACEI and/or ARB use (%), β-blocker use (%), eGFR (mL/min/1.73m2), Calcium channel blocker use (%) and ABI were adjusted. Note: We employed generalized additive models (GAMs) to A generalized additive model (GAM) was used to test the non-linear relationship between DR and HDL-C and to create the smooth curve plots and the p-value for non-linearity in Figures 1, 2, 3, and 4 represents the value of the likelihood ratio test for threshold effects

**Fig. 3:**
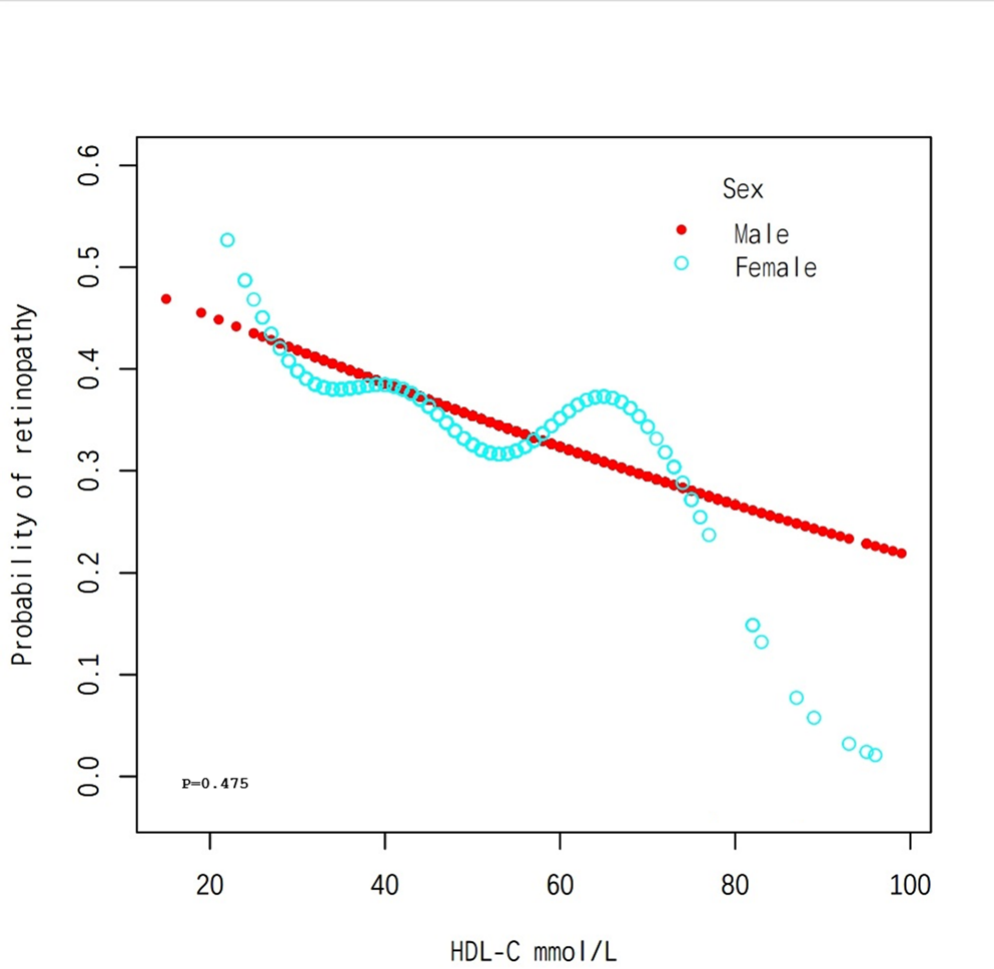
Subgroup analysis stratified by sex. Age, BMI, LDL-C, triglycerides, DBP, Ischaemic Heart Disease, HbA1c(%), Creatine (mg/dL), ACEI and/or ARB use (%), β-blocker use (%), eGFR (mL/min/1.73m2), Calcium channel blocker use (%) and ABI were adjusted. Note: We employed generalized additive models (GAMs) to A generalized additive model (GAM) was used to test the non-linear relationship between DR and HDL-C and to create the smooth curve plots and the p-value for non-linearity in Figures 1, 2, 3, and 4 represents the value of the likelihood ratio test for threshold effects

**Fig. 4:**
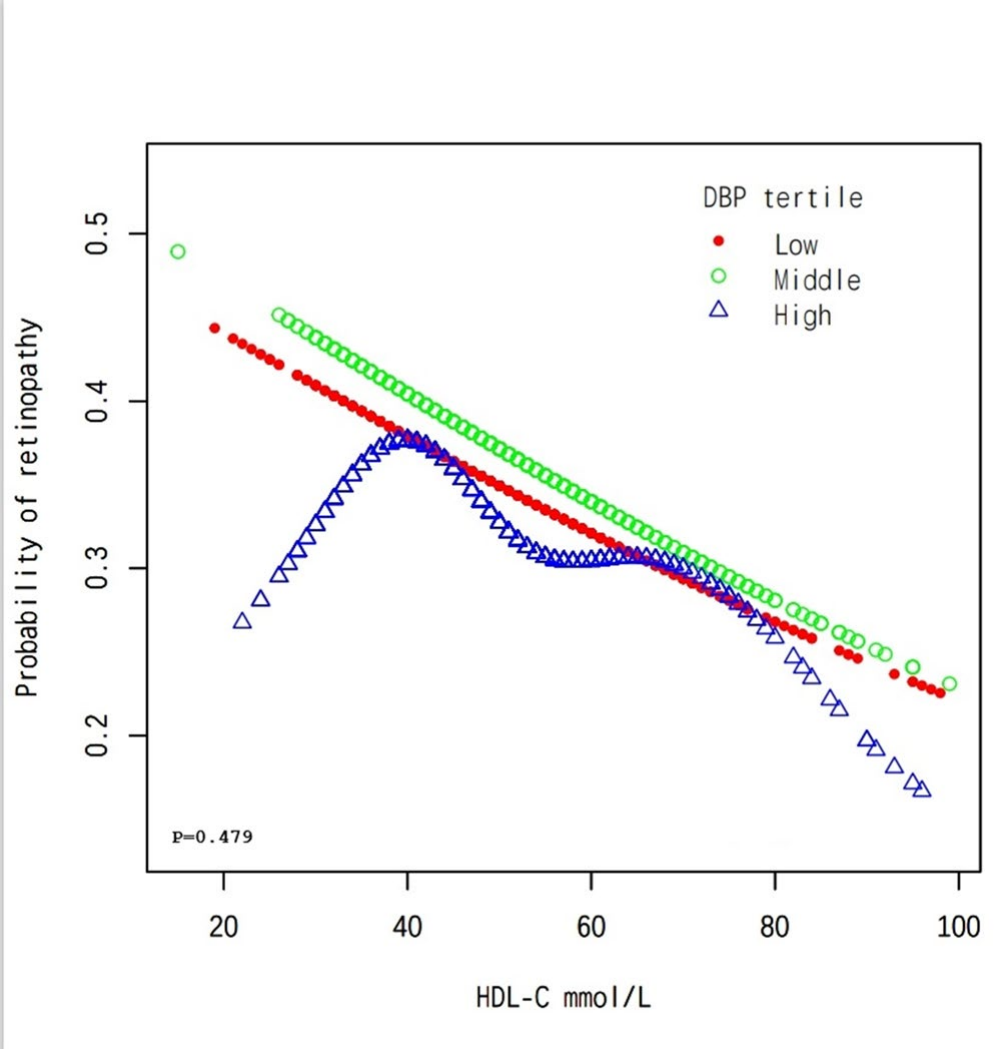
Subgroup analysis stratified by DBP. Sex, age, BMI, LDL-C, triglycerides, Ischaemic Heart Disease, HbA1c(%), Creatine (mg/dL), ACEI and/or ARB use (%), β-blocker use (%), eGFR (mL/min/1.73m2), Calcium channel blocker use (%) and ABI were adjusted. Note: We employed generalized additive models (GAMs) to A generalized additive model (GAM) was used to test the non-linear relationship between DR and HDL-C and to create the smooth curve plots and the p-value for non-linearity in Figures 1, 2, 3, and 4 represents the value of the likelihood ratio test for threshold effects

The overall population analysis showed a linear association between HDL-C levels and the probability of developing DR, indicating that higher HDL-C levels are associated with a reduced likelihood of developing DR (Fig. 1). Subgroup analysis by age revealed consistent patterns, as the probability of DR decreases steadily with increasing HDL-C levels across all age tertiles, with a p-value of 0.517 (Fig. 2). Among female participants, a nonlinear association is observed, suggesting that the relationship between HDL-C and DR may vary by sex, with a p-value of 0.475 (Fig. 3). Finally, the analysis stratified by DBP tertiles showed similar trends, where higher HDL-C levels are associated with lower probabilities of DR, and a p-value of 0.479 remains consistent across different DBP categories (Fig. 4).

## Discussion

In this study, we investigated the Linear relationship between HDL-C and DR. The research utilized a cross-sectional design involving 2001 diabetic patients, which is a large sample size that enhances the reliability of the findings. Our core conclusion indicates a significant negative correlation between HDL-C levels and the risk of DR; specifically, higher HDL-C levels were associated with a reduced risk of DR (odds ratio, 0.91; 95% confidence interval, 0.84 to 0.98, *p* = 0.0327), suggesting a potential protective role of HDL-C in the development of DR. These findings underscore the complex role of lipid metabolism in diabetic complications and provide important insights for clinical practice and future research.

Our findings align with several similar studies while also revealing some differences. Lyons and Popescu et al.‘s [23, 24] cross-sectional study found an inverse association between HDL-C levels and DR risk, consistent with our results [25]. However, not all studies have reached consistent conclusions. Some studies did not find a significant association between HDL-C and DR risk [26, 27]. Morton et al. explored the association of baseline HDL-C with DR and found that HDL-C was not related to DR or any other retinopathy [28]. Xu’s study [6] revealed an inverted U-shaped positive relationship between HDL-C and DR, which a total of 1708 participants from the National Health and Nutrition Examination Survey (NHANES) 2005–2008 were enrolled in the his study. Zhang C’s study [29] showed that he ratio of HDL-C/ApoA is positively correlated with the incidence and Xuan Y’s study [30] found that the relationship was uncertain and required further investigation. The heterogeneity among previous studies may be related to discrepancies in research design, study sample, and control of confounding variables. Notably, we observed variations in the relationship between HDL-C and DR risk across different subgroups. This heterogeneity has also been reported in a study by Li et al. [31]. Using Mendelian randomization, they found that the association between HDL-C and DR risk varied across different genotypes, suggesting that genetic factors may modulate the relationship between HDL-C and DR.

Regarding the potential protective mechanisms of HDL-C against DR, exising research provides some insights. HDL-C has anti-inflammatory and antioxidant properties, which may protect the retina by reducing inflammation and oxidative stress in retinal vessels [32, 33]. Additionally, HDL-C may reduce retinal vascular damage by improving endothelial function and promoting reverse cholesterol transport [34, 35]. In conclusion, our findings support the notion that HDL-C may have a protective effect against DR, while also highlighting the complexity of this relationship. Future prospective and mechanistic studies are needed to further elucidate the exact role of HDL-C in DR development.

The findings of this study provide significant insights for clinical practice, particularly in the relationship between HDL-C and DR. We observed a notable inverse relationship between HDL-C levels and the risk of DR, underscoring the critical role of lipid metabolism in diabetic complications. Compared to existing literature, our study offers more reliable evidence through a larger sample size and comprehensive adjustment for covariates, supporting the potential protective role of HDL-C in the development of DR. This unique contribution may encourage clinicians to consider HDL-C levels as an important biomarker when assessing the risk of DR in diabetic patients. Furthermore, based on our findings, it is advisable for clinical practice to enhance the monitoring of HDL-C levels and implement interventions when necessary to reduce the risk of DR. Future research could further explore the mechanistic role of HDL-C, particularly its effects across different populations, and how optimizing lipid profiles may improve ocular health in diabetic patients.

This study possesses several notable strengths that enhance our understanding of the relationship between HDL-C and DR. Firstly, the cross-sectional design included 2,001 diabetic patients, providing a larger sample size that yields more representative results. Secondly, the data analysis employed multivariate logistic regression models, adequately adjusting for various confounding factors such as sex, age, body mass index (BMI), blood pressure, and glycated hemoglobin (HbA1c), which enhances the reliability of the findings. Additionally, the use of smooth curve fitting and generalized additive models (GAM) to analyze potential nonlinear relationships further elucidated the complex association between HDL-C and DR risk. This methodological approach not only improved the precision of the analysis but also offered new insights into the mechanisms of HDL-C’s effects. Finally, the ethical review and informed consent procedures ensured compliance and ethical integrity, thereby bolstering the credibility of the study’s results.

This study has several limitations that affect the generalizability and external validity of the findings. (1) As a single-center study, all participants were recruited from two hospitals in Taiwan, which limits the generalizability of the results. To enhance external validity, future studies should be conducted across different regions and multiple centers to validate our findings. (2) The primary study population consisted of Chinese individuals, so caution is needed when applying our findings to other ethnic groups. Different ethnicities may exhibit variations in genetics, environment, and lifestyle, which could influence the relationship between HDL-C and DR. (3) As an observational study, while we identified an association between HDL-C and DR, we cannot establish causality. Future prospective studies or randomized controlled trials would be beneficial in further exploring this relationship. Lastly, this study could only adjust for measurable confounding factors and could not control for unmeasurable confounders, such as genetic background and lifestyle habits, which may have a potential impact on the results. (4) The data of this study was collected during the period from 2002 to 2004, which is relatively distant in comparison to the present day and may influence the current applicability of the research findings. In recent years, there have been several changes in diagnostic criteria, treatment approaches, and population characteristics in the medical field, which may impact the mechanisms of DR and its risk factors. Therefore, while our study provides valuable insights, these limitations should be considered when interpreting the findings. (5) We conducted a sensitivity analysis by treating HDL-C as a three-level categorical variable (tertiles: low, middle, high), using the lowest tertile as the reference. The directions of the effect sizes for the middle and highest tertiles were consistent. However, after categorization, the per-group sample sizes decreased; in this study of 2,001 participants, the three tertiles comprised approximately 658, 635, and 699 individuals, respectively (see Table 1). The smaller per-group sizes reduced statistical power, making it more difficult to achieve statistical significance; accordingly, the analyses were underpowered after grouping and the p-values did not reach significance.

## Conclusion

In conclusion, our study provides strong evidence of a significant inverse relationship between HDL-C levels and DR in diabetic patients. The findings indicate that higher HDL-C levels are associated with a reduced prevalence of DR. This research contributes to the growing body of literature emphasizing the role of lipid metabolism in diabetic complications and highlights the importance of monitoring HDL-C levels when evaluating patients for DR.

## Supplementary Information

Below is the link to the electronic supplementary material.

Supplementary File 1 (XLSX 499 KB)

## Data Availability

The data generated and analyzed during this study are available in the Supplementary Information section of the manuscript.

## References

[1] Yau JW, Rogers SL, Kawasaki R, et al. Global prevalence and major risk factors of diabetic retinopathy. Diabetes Care. 2012;35:556–64. 22301125 10.2337/dc11-1909 PMC3322721

[2] Cheung N, Mitchell P, Wong TY. Diabetic retinopathy. Lancet. 2010;376:126–38. 10.1016/S0140-6736(09)62124-3 20580421

[3] Klein R, Klein BE, Moss SE, et al. The Wisconsin epidemiologic study of diabetic retinopathy: XXIII. The twenty-five-year incidence of macular edema. Ophthalmology. 2009;116:327–33. 10.1016/j.ophtha.2008.10.016 PMC2693093 19167079

[4] Yang J, Liu Z. Mechanistic pathogenesis of endothelial dysfunction in diabetic nephropathy and retinopathy. Front Endocrinol. 2022. 10.3389/fendo.2022.816400. 10.3389/fendo.2022.816400 PMC9174994 35692405

[5] Ezhilvendhan K, Sathiyamoorthy A, Prakash BJ, Bhava BS, Shenoy A. Association of Dyslipidemia with Diabetic Retinopathy in Type 2 Diabetes Mellitus Patients: A Hospital-Based Study. J Pharm Bioallied Sci. 2021;13(Suppl 2):S1062–7. 35017930 10.4103/jpbs.jpbs_164_21 PMC8686907

[6] Xu W, Xu X, Zhang M, Sun C. Association between HDL cholesterol with diabetic retinopathy in diabetic patients: a cross-sectional retrospective study. BMC Endocr Disord. 2024;24(1):65. 38730329 10.1186/s12902-024-01599-0 PMC11084017

[7] Wang L, Liu L, Luo H, Wu Y, Zhu L. Correlation Between the Ratio of Uric Acid to High-Density Lipoprotein Cholesterol (UHR) and Diabetic Retinopathy in Patients with Type 2 Diabetes Mellitus:A Cross-Sectional Study. Diabetes Metab Syndr Obes. 18:173-183. 8., Alattas K, Alsulami DW, Alem RH, Alotaibi FS, Alghamdi BA, Baeesa LS. Relation between lipid profile, blood pressure and retinopathy in diabetic patients in King Abdulaziz University hospital: a retrospective record review study. Int J Retina Vitreous. 2022;8(1):20. 10.1186/s40942-022-00366-4 PMC8908615 35264243

[8] Alattas K, Alsulami DW, Alem RH, Alotaibi FS, Alghamdi BA, Baeesa LS. Relation between lipid profile, blood pressure and retinopathy in diabetic patients in King Abdulaziz University hospital: a retrospective record review study. Int J Retina Vitreous. 2022;8(1):20. 35264243 10.1186/s40942-022-00366-4 PMC8908615

[9] Chen SC, Hsiao PJ, Huang JC, Lin KD, Hsu WH, Lee YL, et al. Abnormally low or high ankle-brachial index is associated with proliferative diabetic retinopathy in type 2 diabetic mellitus patients. PLoS ONE. 2015;10(7):e0134718. 26230390 10.1371/journal.pone.0134718 PMC4521755

[10] American Diabetes Association Professional Practice Committee. 2. Diagnosis and classification of diabetes: standards of care in Diabetes-2024. Diabetes Care. 2024;1(Suppl 1):S20–42. 10.2337/dc24-S002 PMC10725812 38078589

[11] Tomiyama H, Yamashina A, Arai T, Hirose K, Koji Y, Chikamori T, et al. Influences of age and gender on results of noninvasive brachial-ankle pulse wave velocity measurement—a survey of 12517 subjects. Atherosclerosis. 2003;166:303–9. 12535743 10.1016/s0021-9150(02)00332-5

[12] Yamashina A, Tomiyama H, Takeda K, Tsuda H, Arai T, Hirose K, et al. Validity, reproducibility, and clinical significance of noninvasive brachial-ankle pulse wave velocity measurement. Hypertens Res. 2002;25:359–64. 12135313 10.1291/hypres.25.359

[13] Vickery S, Stevens PE, Dalton RN, van Lente F, Lamb EJ. Does the id-ms traceable Mdrd equation work and is it suitable for use with compensated Jaffe and enzymatic creatinine assays? Nephrol Dial Transplant. 2006;21:2439–45. 16720592 10.1093/ndt/gfl249

[14] Levey AS, Bosch JP, Lewis JB, Greene T, Rogers N, Roth D, et al. A more accurate method to estimate glomerular filtration rate from serum creatinine: a new prediction equation. Ann Intern Med. 1999;130:461–70. 10075613 10.7326/0003-4819-130-6-199903160-00002

[15] Muntner P, Orroth KK, Mues KE et al. Evaluating a Simple Approach to Identify Adults Meeting the 2018 AHA/ACC Cholesterol Guideline Definition of Very High Risk for Atherosclerotic Cardiovascular Disease. Cardiovasc Drugs Ther. 2022;36(3):475-481. 16. Finsterer J. Neurological implications of cardiac compromise in COVID-19. Am J Cardiovasc Dis. 2023;13(2):43–51. 10.1007/s10557-021-07167-1 PMC8720507 33661432

[16] Finsterer J. Neurological implications of cardiac compromise in COVID-19. Am J Cardiovasc Dis. 2023;13(2):43–51. 37213312 PMC10193247

[17] Roberts PS, Krishnan S, Burns SP, Ouellette D, Pappadis MR. Inconsistent classification of mild stroke and implications on health services delivery. Arch Phys Med Rehabil. 2020;101(7):1243–59. 32001257 10.1016/j.apmr.2019.12.013 PMC7311258

[18] Wang Y, Rodríguez de Gil P, Chen YH, et al. Comparing the performance of approaches for testing the homogeneity of variance assumption in One-Factor ANOVA models. Educ Psychol Meas. 2017;77(2):305–29. 29795915 10.1177/0013164416645162 PMC5965542

[19] Matis G, Birbilis T, Kontogianidis K. Glasgow coma scale and APACHE II system data–are they normally distributed? Chirurgia (Bucur). 2009;104(1):73–8. 19388572

[20] Cheng J, Sun J, Yao K, Xu M, Cao Y. A variable selection method based on mutual information and variance inflation factor. Spectrochim Acta A Mol Biomol Spectrosc. 2022;268:120652. 34896682 10.1016/j.saa.2021.120652

[21] Kim JH. Multicollinearity and misleading statistical results. Korean J Anesthesiol. 2019;72(6):558–69. 31304696 10.4097/kja.19087 PMC6900425

[22] Watkins PJ. Retinopathy. BMJ. 2003;326:924–6. 12714476 10.1136/bmj.326.7395.924 PMC1125830

[23] Popescu T, Moţa M. Dyslipidemia and hypertension in diabetic patients type 2 diabetes and retinopathy[J]. Romanian J Intern Med = Revue Roumaine De Med Interne. 2009;47(3):235–41. 20446438

[24] Chen S, Zhang M, Yang P, Chen S, Zhang M, Yang P, Guo J, Liu L, Yang Z, Nan K et al. Genetic Association between Lipid-Regulating Drug Targets and Diabetic Retinopathy: A Drug Target Mendelian Randomization Study[J]. J Lipids. Genetic Association between Lipid-Regulating Drug Targets and Diabetic Retinopathy: A Drug Target Mendelian Randomization Study[J]. J Lipids. 2024;5(09):5324127. 10.1155/2024/5324127 PMC11098603 38757060

[25] Zhang C, Lin W, Xu Q, et al. Association between high-density lipoprotein cholesterol to Apolipoprotein A ratio and diabetic retinopathy: a cross-sectional study. J Diabetes Complications. 2023;37:108471. 37127002 10.1016/j.jdiacomp.2023.108471

[26] Morton J, Zoungas S, Li Q, Patel AA, Chalmers J, Woodward M, Celermajer DS, Beulens JW, Stolk RP, Glasziou P, et al. Low HDL cholesterol and the risk of diabetic nephropathy and retinopathy: results of the ADVANCE study[J]. Diabetes Care. 2012;35(11):2201–6. 22891258 10.2337/dc12-0306 PMC3476889

[27] Benarous R, Sasongko MB, Qureshi S, Fenwick E, Dirani M, Wong TY, Lamoureux EL. Differential association of serum lipids with diabetic retinopathy and diabetic macular edema[J]. Investig Ophthalmol Vis Sci. 2011;52(10):7464–9. 21862642 10.1167/iovs.11-7598

[28] Lamoureux EL. Differential association of serum lipids with diabetic retinopathy and diabetic macular edema. Invest Ophthalmol Vis Sci. 2011;52(10):7464–9. 21862642 10.1167/iovs.11-7598

[29] Zhang C, Lin W, Xu Q, et al. Association between high-density lipoprotein cholesterol to Apolipoprotein A ratio and diabetic retinopathy: a cross-sectional study. J Diabetes Complications. 2023;37:108471. 37127002 10.1016/j.jdiacomp.2023.108471

[30] Xuan Y, Zhang W, Wang Y, et al. Association between uric acid to HDL cholesterol ratio and diabetic complications in men and postmenopausal women. Diabetes Metab Syndr Obes[J]. 2023;16:167–77. 36760595 10.2147/DMSO.S387726 PMC9869791

[31] Li N, Zhang X, Zhang M, et al. Associations of genetically determined lipid traits and lipid-modifying agents with the risk of diabetic retinopathy: a Mendelian randomization study. Atherosclerosis. 2023;369:9–16. 36827905 10.1016/j.atherosclerosis.2023.02.001

[32] Yang TW. KeSong, rizhenguo, xinlixu, yuanweiwang, jiezhang, qingalexander, Kevin michaelliao, ronglihchen, yucheng.serum high-density lipoprotein cholesterol serves as a prognostic marker for light-chain cardiac amyloidosis[J]. Int J Cardiol, 2021, 325(1). 10.1016/j.ijcard.2020.10.034 33080283

[33] Lenten BJV, Hama SY, Beer FCD, et al. Anti-inflammatory HDL becomes pro-inflammatory during the acute phase response. Loss of protective effect of HDL against LDL oxidation in aortic wall cell cocultures. J Clin Invest. 1995;96(6):2758–67. 8675645 10.1172/JCI118345 PMC185985

[34] Pirillo A, Catapano AL, Norata GD. Biological consequences of dysfunctional HDL. Curr Med Chem. 2019;26(9):1644–64. 29848265 10.2174/0929867325666180530110543

[35] Oleszkiewicz A, Kunkel F, Larsson M, et al. Consequences of undetected olfactory loss for human chemosensory communication and well-being. Philos Trans R Soc Lond B Biol Sci. 2020;375(1800):20190265. 32306872 10.1098/rstb.2019.0265 PMC7209944

